# Primary tumor volume and prognosis for patients with p16-positive and p16-negative oropharyngeal squamous cell carcinoma treated with radiation therapy

**DOI:** 10.1186/s13014-022-02074-7

**Published:** 2022-06-14

**Authors:** Gabriel Adrian, Henrik Carlsson, Elisabeth Kjellén, Johanna Sjövall, Björn Zackrisson, Per Nilsson, Maria Gebre-Medhin

**Affiliations:** 1 Department of Hematology, Oncology, and Radiation Physics, Skåne University Hospital, Lund University, Lund, Sweden; 2 Division of Oncology, Department of Clinical Sciences Lund, Skåne University Hospital, Lund University, Lund, Sweden; 3 Department of Otorhinolaryngology –Head and Neck Surgery, Skåne University Hospital, Lund University, Lund, Sweden; 4 Department of Radiation Sciences, Oncology, Umeå University, Umeå, Sweden; 5 Department of Clinical Sciences, Medical Radiation Physics, Skåne University Hospital, Lund University, Lund, Sweden

## Abstract

**Background:**

The prescribed radiation dose to patients with oropharyngeal squamous cell carcinoma (OPSCC) is standardized, even if the prognosis for individual patients may differ. Easy-at-hand pre-treatment risk stratification methods are valuable to individualize therapy. In the current study we assessed the prognostic impact of primary tumor volume for p16-positive and p16-negative tumors and in relationship to other prognostic factors for outcome in patients with OPSCC treated with primary radiation therapy (RT).

**Methods:**

Five hundred twenty-three OPSCC patients with p16-status treated with primary RT (68.0 Gy to 73.1 Gy in 7 weeks, or 68.0 Gy in 4.5 weeks), with or without concurrent chemotherapy, within three prospective trials were included in the study. Local failure (LF), progression free survival (PFS) and overall survival (OS) in relationship to the size of the primary gross tumor volume (GTV-T) and other prognostic factors were investigated. Efficiency of intensified RT (RT with total dose 73.1 Gy or given within 4.5 weeks) was analyzed in relationship to tumor volume.

**Results:**

The volume of GTV-T and p16-status were found to be the strongest prognostic markers for LF, PFS and OS. For p16-positive tumors, an increase in tumor volume had a significantly higher negative prognostic impact compared with p16-negative tumors. Within a T-classification, patients with a smaller tumor, compared with a larger tumor, had a better prognosis. The importance of tumor volume remained after adjusting for nodal status, age, performance status, smoking status, sex, and hemoglobin-level. The adjusted hazard ratio for OS per cm^3^ increase in tumor volume was 2.3% (95% CI 0–4.9) for p16-positive and 1.3% (95% 0.3–2.2) for p16-negative. Exploratory analyses suggested that intensified RT could mitigate the negative impact of a large tumor volume.

**Conclusions:**

Outcome for patients with OPSCC treated with RT is largely determined by tumor volume, even when adjusting for other established prognostic factors. Tumor volume is significantly more influential for patients with p16-positive tumors. Patients with large tumor volumes might benefit by intensified RT to improve survival.

**Supplementary Information:**

The online version contains supplementary material available at 10.1186/s13014-022-02074-7.

## Background

Squamous cell carcinoma arising in the oropharynx (OPSCC) exemplifies the importance of personalized medicine. Among the more than 100 000 cases annually [1], p16-status (as surrogate for human papillomavirus [HPV]-association) is a watershed for prognosis and is incorporated in the latest edition of the TNM Classification [2, 3]. Patients with p16-positive tumors experience a far better outcome and numerous trials address treatment de-escalation to reduce long-term side effects [4]. On the other hand, the higher failure rates among p16-negative tumors justify trials with intensified treatment to improve survival [5].

Radiation therapy (RT) is often the preferred treatment option for OPSCC, either alone, with chemotherapy (CRT) or in combination with surgery [6]. In theory, a large tumor harbors more clonogenic cells than a small one and in order to obtain cure, all clonogenic cells have to be eliminated. Consequently, tumor volume should be a fundamental prognostic marker and the current T-classification is an attempt to reflect that. Several studies have identified computed tomography (CT)-defined tumor volume to be a prognostic marker in OPSCC treated with RT [7–9]. However, these analyses lack p16-stratification. Recent studies including p16-status to address the importance of tumor volume have provided diverging results [10–13]. Moreover, smoking status, performance status, and hemoglobin (Hb)-levels are established prognostic factors for outcome after RT [13–17]. Their impact in relationship to tumor volume and p16-status has not been studied previously in detail.

We studied a pooled cohort of 654 patients with OPSCC from three clinical trials.

The aim of the work was to quantify the effect of tumor volume on treatment outcome in relation to p16-status and other clinical prognostic factors. In addition, a potential role of intensified RT for high risk-group patients, defined by tumor volume, was investigated.

## Methods

This cohort study provides a pooled analysis of patients with OPSCC treated with primary RT from two randomized clinical trials (ARTSCAN and ARTSCAN III) [18, 19] and one prospective observational study (PET-study) [20]. The main objective of the present study was to determine the impact of primary tumor volume in relationship to p16-status for local failure (LF), progression free survival (PFS) and overall survival (OS). Secondary objectives were to determine the impact of clinical characteristics in relationship to tumor volume and p16-status. The analyses also include exploratory investigations for high-risk group patients as identified by tumor volume-stratification.

The details of the original trials have been previously reported [18–20]. In short, ARTSCAN was a Swedish randomized, controlled phase III study investigating altered fractionated RT, recruiting 1998–2006. The PET-study was a prospective observational single-center study investigating positron emission tomography (PET) for evaluation of neck node response, recruiting 2009–2012. ARTSCAN III was a Swedish randomized, controlled phase III study investigating concomitant cetuximab compared with cisplatin, recruiting 2013–2018. Ethical boards approved all studies. The current study includes all patients with oropharyngeal cancer who completed RT within the three studies, were eligible for evaluation of primary outcome, and had the sizes of the delineated structures available. Individual patient data as assessed in the original studies were pooled and analyzed for the outcomes of interest. The size of the primary gross tumor volume (GTV-T), as delineated by the treating radiation oncologist, was used as the primary tumor volume. These volumes were extracted from the treatment planning system as previously reported for ARTSCAN [21], with Elekta Oncentra MasterPlan, version 4.0 for the PET-study and with Varian Medical Systems, Eclipse, version 15.1 for ARTSCAN III. Patient specific characteristics [age, sex, smoking status, performance status, and Hb-level (before start of RT)] were recorded in the original trials.

### Treatment

All patients received RT as primary treatment. RT was prescribed to 68.0 Gy in 34 fractions, except for the experimental arm in ARTSCAN (1.1 Gy + 2.0 Gy per day, total dose 68.0 Gy) and for the subgroup of T3-4 patients in ARTSCAN III who underwent a second randomization (68.0 Gy or 73.1 Gy in 34 fractions to GTV-T). In the current study, the experimental arm in ARTSCAN and the dose-escalation in ARTSCAN III are termed “intensified RT”. No concomitant drug was used in ARTSCAN, or for the majority of patient in the PET-study (Table 1). ARTSCAN III randomly assigned patients to either concomitant weekly cisplatin 40 mg/m^2^ or cetuximab 400 mg/m^2^ one week before start of RT followed by 250 mg/m^2^/wk.

**Table 1:** Baseline characteristics and treatment specifications stratified by original trial

	ARTSCAN	318 pat		PET-study	92 pat		ARTSCAN III	244 pat		Pooled cohort	
			Available			Available			Available		
Year of treatment			318 (100%)			92 (100%)			244 (100%)		
Range	1998–2006			2009–2012			2013–2018			1998–2018	
Follow-up time in years			318 (100%)			92 (100%)			244 (100%)		
Survival: median (interquartile range)	8.7	(7.1–10.5)		5.4	(5.3–5.9)		3.1	(2.4–4.4)		5.6	(3.8–8.4)
Tumor response: median (interquartile range)	5.3	(5.2–5.5)		5.1	(5.0–5.4)		2.8	(2.1–4.1)		5.1	(3.2–5.4)
Age (y)			318 (100%)			92 (100%)			244 (100%)		
Median (interquartile range)	58	52–64		61	53–64		60.5	54–66		59	(53–65)
Sex			318 (100%)			92 (100%)			244 (100%)		
Male no (%)	234	73.6%		69	75.0%		197	80.7%		500	76.50%
T			318 (100%)			92 (100%)			244 (100%)		
1	56	17.6%		18	19.6%		40	16.4%		114	17.4%
2	130	40.9%		48	52.2%		93	38.1%		271	41.4%
3	74	23.3%		15	16.3%		44	18.0%		133	20.3%
4	58	18.2%		11	12.0%		67	27.5%		136	20.8%
N			318 (100%)			92 (100%)			244 (100%)		
0	68	21.4%		0	0.0%		20	8.2%		88	13.5%
1	65	20.4%		13	14.1%		16	6.6%		94	14.4%
2A	63	19.8%		18	19.6%		17	7.0%		98	15.0%
2B	68	21.4%		48	52.2%		138	56.6%		254	38.8%
2C	29	9.1%		12	13.3%		44	18.0%		85	13.0%
3	25	7.9%		1	1.1%		9	3.7%		35	5.3%
GTV-T (cm^3^)			318 (100%)			92 (100%)			244 (100%)		
Median (interquartile range)	16.4	7.5–30.4		16.4	7.8–28.0		13.2	6.3–24.7		15.4	7.0–28.5
GTV-N (cm^3^)			318 (100%)			91 (99%)			244 (100%)		
Median (interquartile range)	10.4	1.6–22.5		15.7	8.1–26.5		12.4	4.9–21.6		11.6	4.0–22.9
Hb			269 (85%)			80 (87%)			244 (100%)		
Median (interquartile range)	140	131–148		141	134–151		145	137–151		142	133–150
Perfomance status			318 (100%)			92 (100%)			244 (100%)		
WHO 0 or karnofsky 90–100	263	82.7%		89	96.7%		226	92.6%		578	88.4%
WHO ≥ 1 or karnofsky ≤ 80	55	17.3%		3	3.3%		18	7.4%		76	11.6%
Smoker											
Non-smoker	165	51.9%		30	32.6%		73	29.9%		268	41.0%
Previous smoker				24	26.1%		135	55.3%		159	24.3%
Daily smoker*	82	25.8%		38	41.3%		35	14.3%		155	23.7%
Unknown	71	22.3%					1	0.4%		72	11.0%
p16			190 (60%)			91 (99%)			242 (99%)		
p16-positive	141	74.2%		75	82.4%		217	89.7%		433	82.8%
p16-negative	49	25.8%		16	17.6%		25	10.3%		90	17.2%
Radiotherapy			318 (100%)			92 (100%)			244 (100%)		
68 Gy / 34 fx (2 Gy/day)	158	49.7%		92	100%		189	77.5%		439	67.1%
68 Gy / 43 fx (1.1 + 2 Gy/day)	160	50.3%								160	24.5%
73.1 Gy / 68 Gy (2.15 Gy / day)							55	22.5%		55	8.4%
Treatment technique			318 (100%)			92 (100%)			244 (100%)		
Intensity modulated radiotherapy (IMRT)	12	3.8%		92	100%		244	100.0%			
3D-conformal radiotherapy (3DCRT)	306	96.2%									
Concomittant drugs			318 (100%)			92 (100%)			244 (100%)		
No drugs	318	100%		87	94.6%					405	61.9%
Cisplatin				3	3.2%		120	49.2%		123	18.8%
Cetuximab							124	50.8%		124	19.0%
Cisplatin + 5FU, induction				2	2.2%					2	0.3%

*(PET cohort: smoked in the 6 months preceeding RT)

### Event definitions

Treatment failure was defined as the first recurrence, either local, regional or distant (or combinations thereof). Patients with local failure and synchronous regional and/or distant failure were included in the analysis of the local failure. Progression free survival was defined as the time to first recurrence or death by any cause. Time to event was calculated from the first day of RT in all analyses.

### Statistical methods

Univariable Cox proportional hazards regression models were used to analyze the association between patient/tumor characteristics and outcome. In the multivariable model all covariates were included and analyzed according to the complete-case method. Proportional hazard assumptions were tested with Schoenfeld residuals tests. Local failures were illustrated using cumulative incidence, with regional failure, distant failure or death as competing events, and groups compared with Gray’s test. The Fine-Gray model was used in addition to the Cox proportional hazard regressions models to account for regional failures, distant failures or death as competing events when analyzing association between tumor volume and local failures. Event rates for PFS and OS were illustrated with the Kaplan–Meier method and groups compared with the log-rank test. Median follow-up time was determined with the inverse Kaplan–Meier method. The Kruskal–Wallis test was used for comparison of non-parametric data across the three cohorts, and Wilcoxon rank-sum test for non-normal distributed comparison between two groups. Receiver-operator-characteristics (ROC)-analysis was used to dichotomize hemoglobin-levels. To allow an interpretation of the interaction coefficient in the tumor volume analyses in relationship to p16-status, tumor volume was transformed to GTV-T_shift_ = GTV-T − GTV-T_median_. Thereby, the difference in additional relative risk for p16-positive versus p16-negative tumors corresponds to the exponential of the coefficient for the interaction term. All analyses were performed in R version 3.6.3 (R Core Team (2021) R: A language and environment for statistical computing. R Foundation for Statistical Computing, Vienna, Austria. URL https://www.R-project.org/) and the extension packages survival, cmprsk and pROC. Statistical tests were two-sided and p-values ≤ 0.05 were considered significant.

## Results

Five hundred twenty-three patients with OPSCC and known p16-status (82.8% p16-positive, 17.2% p16-negative) were included in the analyses. Additionally, data was available for 131 patients without p16-status. Baseline patient and tumor characteristics are shown in Table 1. For patients with available p16-status median follow-up times for tumor response and overall survival were 4.9 years [interquartile range (IQR) 2.8–5.3 years] and 5.3 years (IQR 3.2–7.3 years), respectively. Patients without p16-status were predominantly found in the ARTSCAN-study, and no differences in outcome were found between patients with or without missing data (Additional File 1: Fig. S1). The three cohorts recruited over two decades, and the proportion of p16-positive tumors increased significantly (*P* < 0.001) over time.

### Treatment outcome

During the follow-up, there were 133 deaths among the 523 patients. A total of 119 patients experienced failure. First appearances of failure were 62 T-failures (30 T-failures, 23 T+N-failures, 5 T+M-failures, 4 T+N+M-failures), 24 N-failures, 28 M-failures, and 5 N+M failures. Forty-two of the 133 deaths occurred without any documented failure. At 5 years, the cumulative incidence of local failure was 13% [95% confidence interval (CI) 10–16], PFS 68% [95% (CI) 64–73] and OS 74% (95% CI 70–78). Survival comparisons across the three cohorts revealed no significant differences (Additional file 1: Fig. S2).

### Analyses of prognostic factors

**Fig. 1:**
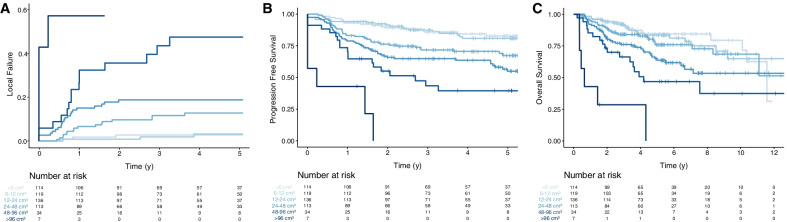
Impact of tumor volume and outcome after radiation therapy for 523 patients with oropharyngeal squamous cell carcinoma and available p16-status. Patients were stratified by tumor volume-doublings (< 6 cm^3^ [lightest blue line], 6–12 cm^3^, 12–24 cm^3^, 24–48 cm^3^, 48–96 cm^3^, and > 96 cm^3^ [darkest blue line]). Illustrations of cumulative incidence of local failure **A**, progression free survival **B** and overall survival **C**

Tumor volume was the strongest prognostic factor for LF, PFS and OS in the univariable cox-regression analyses as reflected in the likelihood-ratio test (Table 2 and latter part of Table 3). The importance of tumor volume for LF, PFS, and OS was statistically significant within each T-classification for all endpoints. To illustrate the impact of tumor volume on treatment outcome, patients were stratified into six groups by tumor volume-doublings. Clear separations between the six volume-groups were evident, with similar results for LF, PFS and OS (Fig. 1A–C).

**Table 2:** Univariable Cox-regressions of tumor volume (GTV-T) for local failure, progression free survival and overall survival for all patients or separately within each T-classification or by p16-stratification. HR: Hazard Ratio, lr-test: the difference in -2 log likelihood between the null model and the full model

	Local failure				Progression free survival				Overall survival			
	HR	CI	*p*	lr-test	HR	CI	*p*	lr-test	HR	CI	*p*	lr-test
*Without p16-stratification (all pat)*												
GTVT-volume (per cm^3^)	1.030	1.024–1.037	** < 0.001**	64	1.025	1.020–1.029	** < 0.001**	76	1.025	1.020–1.030	** < 0.001**	70.2
GTVT-volume within T1 (per cm^3^)	1.161	1.001–1.338	**0.04**	3.6	1.081	1.018–1.150	**0.01**	5.2	1.104	1.034–1.179	**0.003**	7.14
GTVT-volume within T2 (per cm^3^)	1.041	1.013–1.069	**0.003**	5.8	1.028	1.012–1.045	** < 0.001**	9.1	1.021	1.002–1.040	**0.03**	4.0
GTVT-volume within T3 (per cm^3^)	1.024	1.007–1.042	**0.005**	5.9	1.023	1.010–1.035	** < 0.001**	10	1.020	1.008–1.032	** < 0.001**	8.5
GTVT-volume within T4 (per cm^3^)	1.018	1.009–1.028	** < 0.001**	12	1.015	1.007–1.023	** < 0.001**	12	1.012	1.003–1.021	**0.004**	7.4
*With p16-stratification*												
p16 + : GTVT-volume (per cm^3^)	1.038	1.027–1.049	** < 0.001**	34	1.028	1.020–1.036	** < 0.001**	36	1.024	1.015–1.033	** < 0.001**	22.1
p16-: GTVT-volume (per cm^3^)	1.019	1.010–1.028	** < 0.001**	13	1.014	1.006–1.022	** < 0.001**	9.2	1.018	1.010–1.027	** < 0.001**	13.2

Bold denotes statistical significance (*P* < 0.05)

**Table 3:** Univariable and multivariable Cox-regressions for local failure, progression free survival and overall survival

	Local failure							Progression free survival							Overall survival						
	univariable				multivariable			univariable				multivariable			univariable				multivariable		
	HR	CI	p	lr-test	HR	CI	p	HR	CI	p	lr-test	HR	CI	p	HR	CI	p	lr-test	HR	CI	p
*GTVT per cm*^*3*^* (p16-negative [interaction analyses]) †*	1.020	1.012–1.029	** < 0.001**		1.020	1.009–1.031	** < 0.001**	1.015	1.007–1.023	** < 0.001**		1.011	1.003 – 1.020	**0.011**	1.019	1.011–1.028	** < 0.001**		1.013	1.003 – 1.022	**0.008**
*p16-status (p16-positive vs. p16-negative)*	0.216	0.117–0.401	** < 0.001**		0.256	0.122 – 0.538	** < 0.001**	0.255	0.175–0.371	** < 0.001**		0.269	0.171 – 0.424	** < 0.001**	0.222	0.150–0.329	** < 0.001**		0.241	0.147 – 0.394	** < 0.001**
*GTVT: p16 per cm*^*3*^* §*	1.017	1.003–1.031	**0.016**		1.016	1.001 – 1.032	**0.043**	1.012	1.002–1.023	**0.025**		1.016	1.003 – 1.028	**0.014**	1.004	0.993–1.016	0.47		1.010	0.997 – 1.024	0.144
Age (per y)	1.040	1.010 – 1.070	**0.009**	14	1.011	0.974 – 1.050	0.56	1.050	1.030 – 1.069	** < 0.001**	26	1.032	1.009 – 1.056	**0.007**	1.070	1.049 – 1.091	** < 0.001**	44	1.044	1.019 – 1.069	** < 0.001**
Sex (female vs. male)	1.294	0.741 – 2.262	0.37	0.8	1.348	0.639 – 2.841	0.43	1.127	0.775 – 1.638	0.53	0.4	1.145	0.716 – 1.833	0.57	0.987	0.662 – 1.471	0.95	0	0.937	0.556 – 1.580	0.81
Smoking (current or previous vs. non-smokers)	2.981	1.603 – 5.542	**0.001**	14	1.854	0.918 – 3.743	0.09	2.312	1.582 – 3.380	** < 0.001**	21	1.410	0.927 – 2.144	0.11	2.431	1.617 – 3.655	** < 0.001**	20	1.326	0.843 – 2.086	0.22
Performance status: WHO ≥ 1/ karnofsky ≤ 80 vs. better	4.052	2.340 – 7.017	** < 0.001**	20	1.430	0.697 – 2.934	0.33	3.493	2.405 – 5.073	** < 0.001**	33.8	1.228	0.762 – 1.980	0.40	3.853	2.633 – 5.639	** < 0.001**	38	1.526	0.925 – 2.518	0.10
Hb, > 130 vs ≤ 130	0.474	0.267 – 0.842	**0.01**	5.8	1.149	0.533 – 2.478	0.72	0.485	0.333 – 0.707	** < 0.001**	13	0.651	0.412 – 1.029	0.07	0.479	0.322 – 0.713	** < 0.001**	12	0.599	0.366 – 0.981	**0.042**
N-stage				5.5							15							11			
N0 vs N1-N2b[ref]	2.058	1.047 – 4.043	**0.036**		1.047	0.424 – 2.584	0.92	1.533	0.942 – 2.494	0.086		0.833	0.445 – 1.559	0.57	1.389	0.830 – 2.322	0.21		0.621	0.302 – 1.277	0.20
N2c-N3 vs N1-N2b[ref]	1.697	0.929 – 3.102	0.085		1.464	0.770 – 2.784	0.25	2.075	1.434 – 3.003	** < 0.001**		1.657	1.097 – 2.505	**0.017**	2.011	1.359 – 2.978	** < 0.001**		1.736	1.112 – 2.710	**0.015**

Bold denotes statistical significance (*P* < 0.05)

*HR* Hazard Ratio, *CI* 95% Confidence Interval, *GTVT* Primary gross tumor volume, *lr-test* The difference in -2 log likelihood between the null model and the full model

^†^: The Cox regression analyses were performed including an interaction term between GTVT and p16-status (GTVT*p16-status). The hazard ratio (HR) for GTVT volume is for p16-negative patients

^§^: “GTVT: p16 per cm^3^” corresponds to the additional relative risk per cm^3^ GTVT volume for p16-positive compared with p16-negative patients at the median GTV-T volume (15 cm^3^). For example, for LF, the univariable risk per cm^3^ for a p16-positive patient would be 1.020*1.017 = 1.037

Patients with p16-positive tumors had a more favorable outcome, with cumulative incidence of LF, PFS, and OS at 5 years of 9% (95% CI 6–12), 76% (95% CI 72–81), and 83% (95% CI 79–87) compared with 29% (95% CI 20–39), 34% (95% CI 25–46), and 36% (95% CI 27–48) for p16-negative patients. The negative prognostic impact of tumor volume was higher for patients with p16-positive tumors, and the risk per cm^3^ increase in tumor volume for LF, PFS-event, or death (OS) were 3.8% (95% CI 2.7–4.9), 2.8% (95% CI 2.0–3.8), and 2.4% (1.5–3.3), respectively. Corresponding figures for patients with p16-negative tumors patients were 1.9% (95% CI 1.0–2.8), 1.4% (0.6–2.2), and 1.8% (95% CI 1.0–2.7) (Table 2). Significant interactions between tumor volume (GTV-T_shift_) and p16-status was found for LF (*P* = 0.016) and PFS (*P* = 0.025). Hence, the negative prognostic impact of an increase in tumor volume was significantly higher in p16-positive tumors. For LF, an increase in tumor volume from 15 cm^3^ (GTV-T_median_) to 16 cm^3^ increased the relative risk 1.7 percentage points (95% CI 0.3–3.0) more in p16-positive compared with p16-negative tumors (Table 3). Corresponding figure for PFS was 1.2 percentage points (95% CI 0.2–2.3). In a Fine-Gray model to account for competing events, similar results for LF were obtained [p16-positive 3.7% (95% CI 2.7–4.7, *P* =  < 0.001) for each cm^3^ increase in tumor volume, and for p16-negative 1.8% (95% CI 0.7–2.9, *P* = 0.0015)]. p16-positive tumors were significantly smaller (median 13 cm^3^, IQR 7–26) compared with p16-negative tumors (median 20 cm^3^, IQR 10–35), *P* < 0.001.

Besides tumor volume and p16-status, we found that age, smoking status, performance status, and Hb were also significant factors for LF, PFS and OS in univariable analyses (Table 3 and Additional File 1: Fig. S3). Advanced N-stage (N2c-N3) was significant for PFS and OS. In the multivariable analyses, tumor volume and p16-status remained strongly significant factors for LF, PFS and OS, and the interaction term (p16-status * GTV-T_shift_) was significant for LF and PFS (Table 3). In addition, hemoglobin and age were significant for OS in multivariable regressions. Although highly significant in univariable analysis, no significant impact of smoking status or performance status remained in the multivariable model. Advanced N-stage remained negative prognostic factors for PFS and OS in multivariable analyses. The adjusted risk per cm^3^ increase in tumor volume for LF, PFS and OS were 3.6% (95% CI 0.9–6.4), 2.7% (95% CI 0.6–4.8), and 2.3% (95% CI 0–4.7) for patients with p16-postive tumors. Corresponding figures for patients with p16-negative tumors were 2.0% (95% CI 0.9–3.1), 1.1% (95% CI 0.3–2.0), and 1.3% (95% 0.3–2.2).

### Tumor volume compared with T-classification

Side-by-side comparisons between T-classification and tumor volume are shown in Additional File 1: Fig. S4. For LF, the hazard ratio (HR) for T4 versus T1 was 21 [95% CI 5–88] compared with HR 65 [95% CI 12–344] for the largest compared with the smallest volume bin (as defined above). Tumor volumes within T-classifications reveal a large overlap between T-classifications (Additional File 1: Fig. S5). A tumor volume ≤ 19 cm^3^ identified the same number of patients as the T1-T2 classifications (385 patients). Despite including all T-classifications, this tumor volume-defined low-risk group resulted in similar outcomes compared with T1-T2-classifications for LF, PFS and OS (Additional File 1: Fig. S6).

### Analyses of tumor volume thresholds

**Fig. 2:**
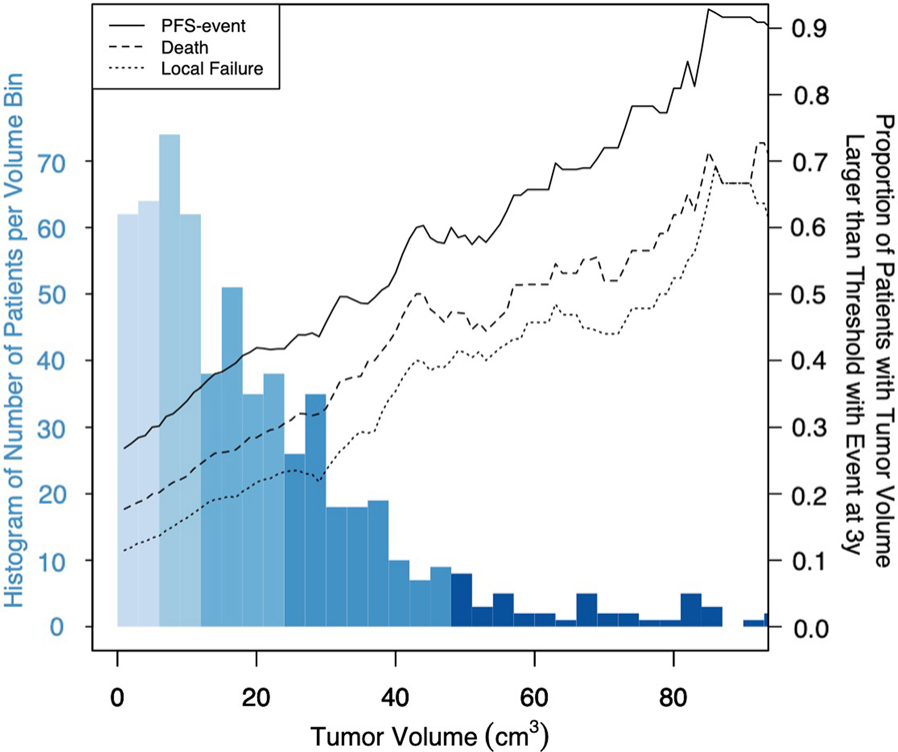
Illustration of different volume thresholds and its impact on outcome. Black lines show the proportion of patients above the given threshold (tumor volume on the *x*-axis) who experience an event within 3 years from treatment for the three endpoints studied (local failure (dotted line), progression free survival (PFS, solid line), and overall survival (dashed line)). Patients without event and shorter follow-up than 3 years were included in the denominator. Histogram denotes the number of patients in each volume bin, color-coded according to Fig. 1

The effect of different tumor volume thresholds for LF, PFS, and OS is illustrated in Fig. 2. With increasing tumor volume, a continuous increase in the number of events (within 3 years from treatment) for all endpoints was seen. Receiver-operator-characteristic (ROC)-analyses between tumor volume and local failures are shown in Additional File 1: Fig. S7.

### High-risk group and intensified RT

**Fig. 3:**
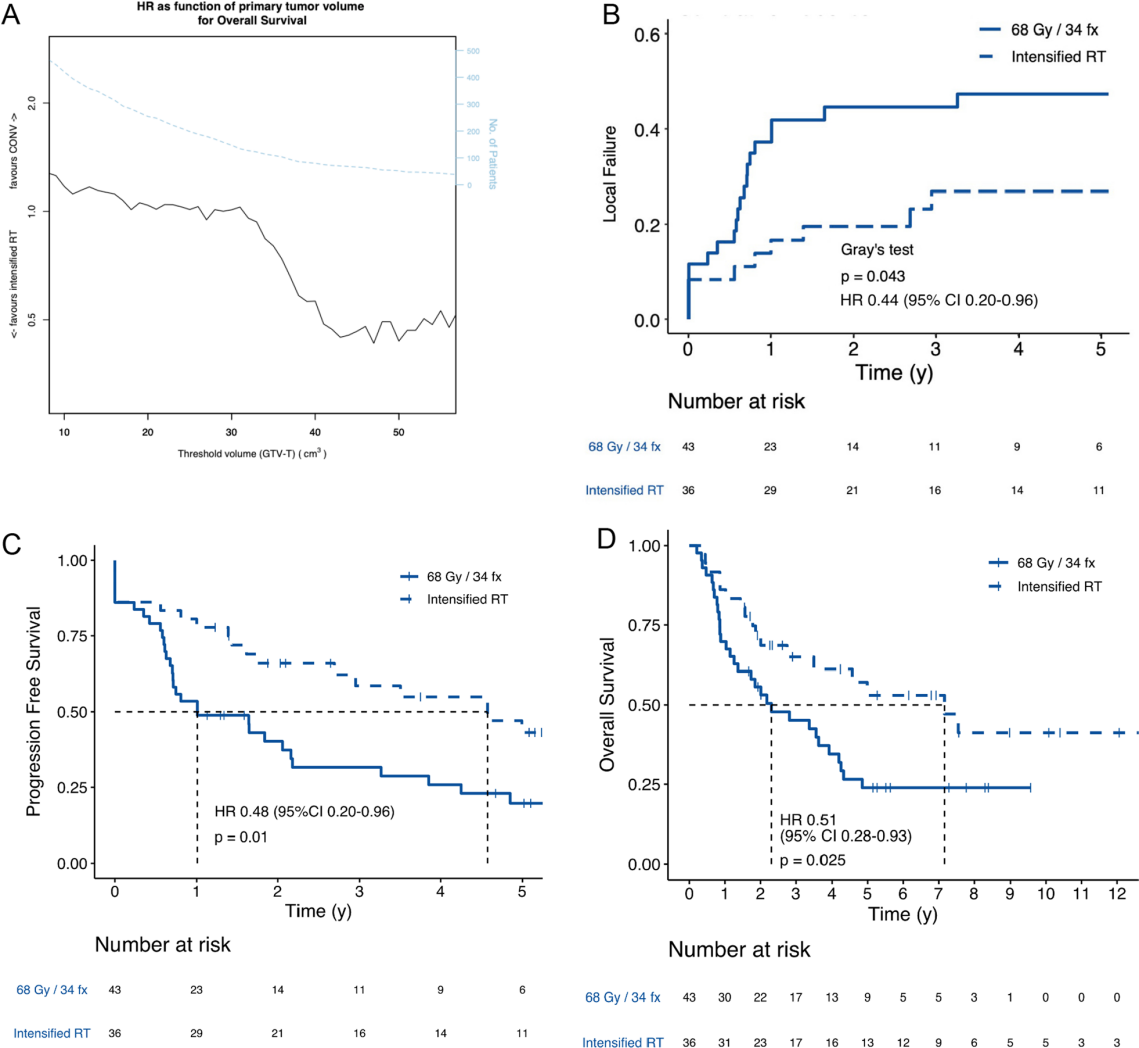
Efficacy of intensified RT to mitigate the negative prognosis associated with large tumor volumes. Illustration of the impact of tumor volume (x-axis) and efficacy of intensified RT (either 1.1 Gy + 2.0 Gy per day, total dose 68.0 Gy or 2.15 Gy per day, total dose 73.1 Gy) compared with conventional RT (CONV, 2.0 Gy per day, total dose 68.0 Gy) (Fig. 3A). The hazard ratio (HR, black solid line) for patients with a tumor volume larger than the indicated volume on the *x*-axis, was investigated using univariable cox-regression with overall survival as endpoint. The number of patients included in each analysis is shown in the upper part (light blue, dashed line). Based on **A**, a cut-off of GTV-T > 40 cm^3^ was chosen for exploratory post-hoc analyses. For high-risk patients (GTV-T > 40 cm^3^) the efficacy of intensified RT is illustrated with regards to the cumulative incidence of local failures (**B**), progression free survival (**C**) and overall survival (**D**). Stratification of patients based on T-classifications revealed no significant effects of intensified RT (Additional File 1: Fig. S8). CI: Confidence Interval

Exploratory analyses revealed statistically significant interactions between tumor volume and intensified RT found for all studied endpoints (LF *P* = 0.009; PFS, *P* = 0.012; OS, *P* = 0.038). The impact of tumor volume-threshold for the comparison was investigated and revealed an increasing efficacy of intensified RT with increasing tumor volume (Fig. 3A). The effect seemed to plateau at a primary tumor volume of 40 cm^3^, which was used for further exploratory analyses. In this group of 79 patients intensified RT significantly improved LF, PFS, and OS (Fig. 3B–D). T-classifications revealed no significant effects of intensified RT (Additional File 1: Fig. S8).

## Discussion

In this study, we have found that primary tumor volume and p16-status are highly influential factors for outcome after primary RT for patients with OPSCC. We show that tumor volume is an independent prognostic marker for LF, PFS and OS. The negative impact of increasing tumor volume is significantly more important for patients with p16-positive compared with p16-negative tumors. Within a given T-classification, patients with a small, compared with a large tumor, have a better prognosis. The results also indicate that intensified RT may mitigate the negative prognostic impact of a large tumor volume.

To our knowledge, this study constitutes the largest OPSSC-cohort treated with primary RT, includes p16-status, and benefits from a long follow-up within prospective trials. The relationship between tumor volume and outcome is congruent to other OPSCC-studies without p16-stratification [7–9] and for head and neck cancer squamous cell carcinoma (HNSCC) in general [22–27]. A pre-clinical HNSCC-model [28] and mathematical modeling of tumor control probability (TCP) also support the impact of tumor volume on outcome [29–31]. However, some earlier OPSCC-studies have only found weak or no relationship between primary tumor volume and outcome [32–35]. Results from studies including p16-status are few and diverging [10–13]. The conflicting results might in part be due to the typically fewer number of included patients. Davis et al*.* studied 51 patients with p16-positive OPSCC, and could not relate disease-free survival to primary tumor volume [11]. In a cohort of 91 patients, Carpén et al*.* found a relationship between primary tumor volume and OS for p16-negative but not p16-positive cases [10]. Our current findings do, however, strongly suggest that CT-determined tumor volume per se is a fundamental prognostic factor for outcome after RT, and its importance is even more pronounced for patients with p16-positive tumors.

In addition to CT-determined tumor volume, metabolic active tumor volume determined by 18F-fluorodeoxyglucose (FDG)-PET has been shown to be prognostic for loco-regional control in p16-positive OPSCC. [36, 37] A large hypoxic tumor volume, determined with PET-tracers such as fluoromisonidazole (FMISO) or fluoroazomycin-ara-binofuranoside (FAZA), also correlates to worse prognosis [38, 39], although the relationship between hypoxia and tumor volume is unclear [40, 41]. Hypoxic gene signatures has been shown to be prognostic for patients with small HNSCC tumors, and a significant interaction between tumor volume and hypoxic gene signatures was found [12]. When comparing different gene signature profiles in HPV-negative HNSCC, tumor volume was found to be the most important factor for OS [41].

The negative impact of a large tumor volume could partly be mitigated by intensified RT in our exploratory analyses. This finding is in line with the EORTC 22791-trial where local control at five years for patients with T3-tumors was more than doubled in the experimental intensified arm compared with standard treatment, whereas patients with T2-tumors had no benefit [42]. Moreover, the EORTC 22851-trial and the CHART-trial showed that experimental, intensified treatment was more advantageous for patients with higher T-classification [43, 44]. The interaction between tumor volume and fractionation schedules is further supported by similar findings for lung cancer [45, 46]. Zhao et al*.* found that patients with large (> 52 cm^3^) lung tumors benefitted from an increased radiation dose, whereas patients with smaller (≤ 52 cm^3^) tumors did not [45]. Soliman et al*.* used a Cox-Regression model when analyzing patients in the lung cancer CHARTWEL-trial and could demonstrate that tumor volume significantly increased the risk of LF in the standard arm but not in the experimental arm, suggesting an increased efficacy for the intensified treatment for patients with larger tumors [46]. The addition of chemotherapy also improves outcome, as shown by the MACH-NC-meta analysis [47]. In the current analysis, patients from the ARTSCAN III-cohort received concurrent cetuximab or cisplatin, and the proportion of drugs was similar in the 68.0 and 73.1 Gy group. The most effective combination of chemotherapy and intensified RT is outside the scope of the present analysis.

The current study confirms the importance of Hb. Contrary to recent findings for HNSCC, where adjusting for CT-determined tumor volume diminished the importance of Hb[13], our findings indicate that Hb > 130 g/L is beneficial for OS also in multivariate analyses. In contrast to earlier multivariable regression models smoking status and performance status were only prognostic in univariable analyses [15, 16]. It should, however, be noted that pack-years was not available, instead smoking status was analyzed for never-smokers vs. previous or current smokers. Age was not prognostic for local failure in the current multivariable model. These results indicate that older patients are not of higher risk for treatment failures, although endpoints involving death for unequivocal reasons are related to age. The improved outcome for patients with p16-postive tumors (adjusted HR for OS 0.24 [95% CI 0.15–0.40]) is comparable to previous reports [2, 16]. Patients with p16-positive tumors typically present with a smaller primary tumor [10, 48, 49], and in the current study the p16-positive tumors were significantly smaller compared with p16-negative. The increased risk of failure for patients with large tumor volumes could thereby partly be attributed to higher proportions of p16-negative tumors. However, in the multivariable model with an interaction analysis of p16-status and tumor volume, the impact of tumor volume remained and was significantly more important for p16-postive tumors.

This study has some limitations. Firstly, being a post-hoc analysis, the solidity of the findings is weakened, although the data originates from prospective trials with multi-center participants and long follow-up, which strengthens the results. The three cohorts differed in the prescribed treatment leading to heterogeneity in the studied population. Patients in the ARTSCAN-trial and the PET-study (except for five patients) were treated with radical RT, and a proportion of these patients would have received concurrent chemotherapy according to current clinical guidelines [6]. The volume assessments are entirely CT-based, and the increased soft-tissue discrimination by magnetic resonance imaging [50] or the importance of metabolic active or hypoxic volume as determined by PET cannot be assessed. Moreover, the volume delineations are based on the planning CT-scans, and not diagnostic pre-biopsy scans. The volume cut-offs used in the study must thus be handled cautiously. The benefit of intensified RT for patients with large tumors is an exploratory post-hoc finding, which limits its validity. However, the data originate from two trials where the allocation of patients to the fractionation schedules was randomized. The relevance for other HNSCC-subsites must be studied separately, as the current findings solely relate to OPSCC.

## Conclusion

In this large cohort of patients with oropharyngeal cancer treated with RT, we have shown that primary tumor volume and p16-status are highly influential factors for local failure, progression free survival and overall survival. The importance of tumor volume is even more pronounced in patients with p16-positive tumors, compared with p16-negative. Tumor volume can be used to identify high-risk groups, where intensified treatment might increase survival. Future studies investigating personalized therapy based on risk-group stratification are indicated.

## Supplementary Information

**Additional File 1: Fig. S1.** Overall survival for patients with complete (dark blue line) or incomplete (light blue line) data within the ARTSCAN-cohort. **Fig. S2.** Progression Free Survival (A and C) and Overall Survival (B and D) stratified by study cohort (ARTSCAN, PET-study, and ARTSCAN III) and separated for p16-status (p16-positive in A and B, p16-negative in C and D). **Fig. S3.** The prognostic impact of age, performance status, smoking status, Hb, p16-status and T-classification for cumulative incidence of local failure, progression free survival and overall survival. **Fig. S4.** Comparison of T-classification (left panel) and tumor volume-stratification (based on tumor volume doublings, right panel) for local failure (A), progression free survival (B) and overall survival (C) of the 654 patients in the whole cohort. **Fig. S5.** Violin plot illustrating delineated tumor volume (GTV-T) grouped by T-classification. Each dot represents a delineated tumor. Color corresponds to the volume bins in Fig 1. **Fig. S6.** A low-risk group defined by tumor volume <19 cm^3^ classified the same number of patients as with T1–T2-classification. In this group of 387 patients all T-classifications were represented (T1: 107 patients, T2: 198 patients, T3: 44 patients, T4: 38 patients). Cumulative incidence of local failure, PFS and OS at 5 years were 5% (95% CI 3-8), 78% (95% CI 74–83), and 82% (95%CI 78–86), respectively. This definition of a low-risk group (panel A–C, dark blue line) was compatible to patients with T1-2 classification regarding outcome [panel B–D, light blue line, 385 patients, with corresponding LF, PFS and OS at 5 years of 4% (95% CI 3–7), 79% (95% CI 75–83), and 84% (95% CI 80-88), respectively]. **Fig. S7.** Receiver-operator-characteristic (ROC)-analysis was used to determine the relationship between tumor volume and local tumor failures. Area under the curve (AUC) for tumor volume to predict T-failure within 3 years was 0.76 (95% CI 0.71–0.82) (blue line). By combining tumor volume and p16-status as predictors, AUC increased to 0.81 (95% CI 0.75–0.87) (black line). **Fig. S8.** Exploratory post-hoc analyses of patients with T3-4- (panel A and B) or T4-tumors (panel C and D) and outcome after intensified radiotherapy (either 1.1Gy+2.0 Gy per day, total dose 68.0 Gy or 2.15 Gy per day, total dose 73.1 Gy) compared with conventional fractionation (2.0 Gy per day, total dose 68.0 Gy). For patients with T3-4 tumors, progression-free survival (panel A) with intensified RT (dotted line) was median 5.0 years (95% CI 4.6-NA) compared with 4.1 years (95% CI 2.3-NA) for standard RT (solid line). Corresponding numbers for overall survival (B) were 8.3 years (95% CI 4.9-NA) and 6.3 years (4.2-NA), respectively. For patients with T4 tumors, progression-free survival (panel C) with intensified RT (dotted line) was median 4.9 years (95% CI 2.7-NA) compared with 2.2 years (95% CI 1.6-NA) for standard RT (solid line). Corresponding numbers for overall survival (D) were 7.6 years (95% CI 4.9-NA) and 3.6 years (2.8–6.6), respectively.

## Data Availability

Research data are not available at this time.

## Abbreviations

CI: Confidence interval
CRT: Chemo-radiation therapy
CT: Computed tomography
GTV-T: Primary gross tumor volume
Hb: Hemoglobin
HNSCC: Head and neck squamous cell carcinoma
HPV: Human papillomavirus
HR: Hazard ratio
IQR: Interquartile range
LF: Local failure
OPSCC: Oropharyngeal squamous cell carcinoma
OS: Overall survival
PET: Positron emission tomography
PFS: Progression-free survival
ROC: Receiver-operator-characteristics
RT: Radiation therapy
TCP: Tumor control probability
