# Supplementary material for: Primary tumor volume and prognosis for patients with p16-positive and p16-negative oropharyngeal squamous cell carcinoma treated with radiation therapy
Source: Radiat Oncol. 2022 Jun 14;17:107. doi: 10.1186/s13014-022-02074-7 (PMC9195357; doi:10.1186/s13014-022-02074-7)
Supplement: Supplementary file 1 — Additional File 1: Fig. S1. Overall survival for patients with complete (dark blue line) or incomplete (light blue line) data within the ARTSCAN-cohort. Fig. S2. Progression Free Survival (A and C) and Overall Survival (B and D) stratified by study cohort (ARTSCAN, PET-study, and ARTSCAN III) and separated for p16-status (p16-positive in A and B, p16-negative in C and D). Fig. S3. The prognostic impact of age, performance status, smoking status, Hb, p16-status and T-classification for cumulative incidence of local failure, progression free survival and overall survival. Fig. S4. Comparison of T-classification (left panel) and tumor volume-stratification (based on tumor volume doublings, right panel) for local failure (A), progression free survival (B) and overall survival (C) of the 654 patients in the whole cohort. Fig. S5. Violin plot illustrating delineated tumor volume (GTV-T) grouped by T-classification. Each dot represents a delineated tumor. Color corresponds to the volume bins in Fig 1. Fig. S6. A low-risk group defined by tumor volume <19 cm3 classified the same number of patients as with T1–T2-classification. In this group of 387 patients all T-classifications were represented (T1: 107 patients, T2: 198 patients, T3: 44 patients, T4: 38 patients). Cumulative incidence of local failure, PFS and OS at 5 years were 5% (95% CI 3-8), 78% (95% CI 74–83), and 82% (95%CI 78–86), respectively. This definition of a low-risk group (panel A–C, dark blue line) was compatible to patients with T1-2 classification regarding outcome [panel B–D, light blue line, 385 patients, with corresponding LF, PFS and OS at 5 years of 4% (95% CI 3–7), 79% (95% CI 75–83), and 84% (95% CI 80-88), respectively]. Fig. S7. Receiver-operator-characteristic (ROC)-analysis was used to determine the relationship between tumor volume and local tumor failures. Area under the curve (AUC) for tumor volume to predict T-failure within 3 years was 0.76 (95% CI 0.71–0.82) (blue line). By combining tumo [file 13014_2022_2074_MOESM1_ESM.docx]

# Supplementary

## Supplementary Fig S1

Overall survival for patients with complete (dark blue line) or incomplete (light blue line) data within the ARTSCAN-cohort.

## Supplementary Fig S2.

Progression Free Survival (A and C) and Overall Survival (B and D) stratified by study cohort (ARTSCAN, PET-study, and ARTSCAN III) and separated for p16-status (p16-positive in A and B, p16-negative in C and D).

## Supplementary Fig S3

The prognostic impact of age, performance status, smoking status, Hb, p16-status and T-classification for cumulative incidence of local failure, progression free survival and overall survival.





##

## Supplementary Fig S4

Comparison of T-classification (left panel) and tumor volume-stratification (based on tumor volume doublings, right panel) for local failure (A), progression free survival (B) and overall survival (C) of the 654 patients in the whole cohort.

A)


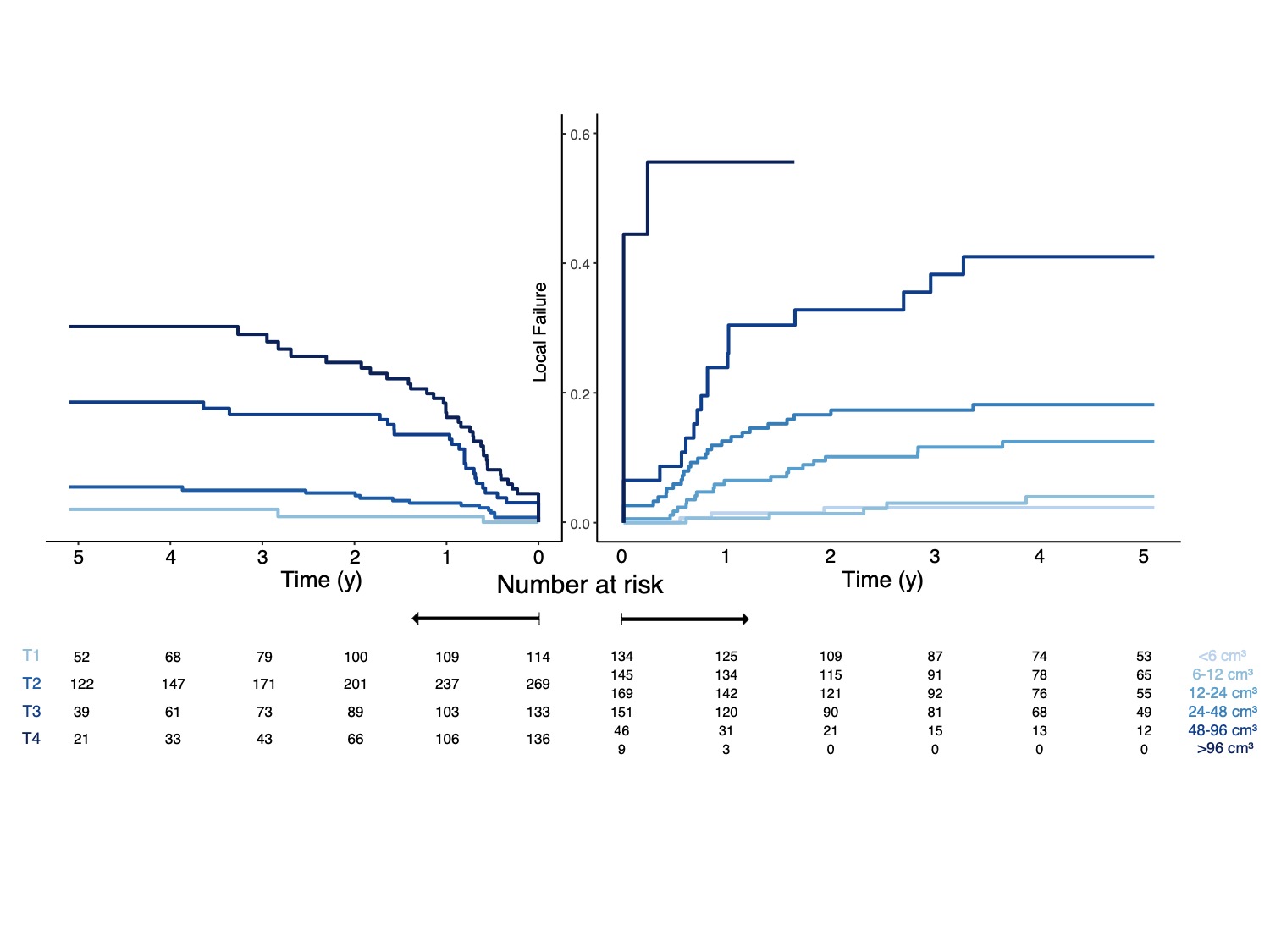


B)

C)

## Supplementary Fig S5

Violin plot illustrating delineated tumor volume (GTV-T) grouped by T-classification. Each dot represents a delineated tumor. Color corresponds to the volume bins in Fig 1.

##
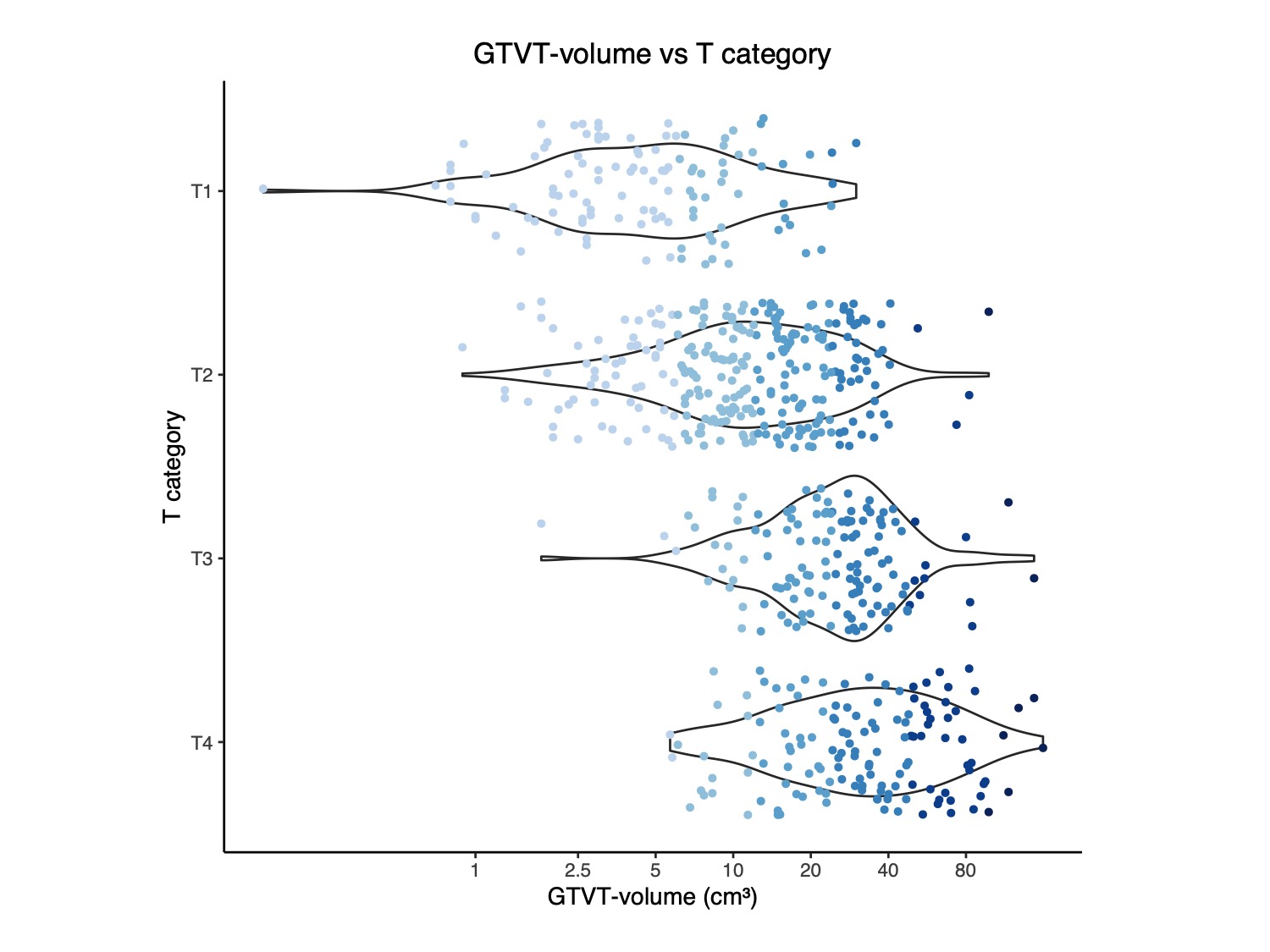


## Supplementary Fig S6

A low-risk group defined by tumor volume <19 cm^3^ classified the same number of patients as with T1-T2-classification. In this group of 387 patients all T-classifications were represented (T1: 107 patients, T2: 198 patients, T3: 44 patients, T4: 38 patients). Cumulative incidence of local failure, PFS and OS at 5 years were 5% (95% CI 3-8), 78% (95% CI 74-83), and 82% (95%CI 78-86), respectively. This definition of a low-risk group (panel A-C, dark blue line) was compatible to patients with T1-2 classification regarding outcome (panel B-D, light blue line, 385 patients, with corresponding LF, PFS and OS at 5 years of 4% [95% CI 3-7], 79% [95% CI 75-83], and 84% [95% CI 80-88], respectively).

A)

B)

C)

##

## Supplementary Fig S7

Receiver-operator-characteristic (ROC)-analysis was used to determine the relationship between tumor volume and local tumor failures. Area under the curve (AUC) for tumor volume to predict T-failure within 3 years was 0.76 (95% CI 0.71-0.82) (blue line). By combining tumor volume and p16-status as predictors, AUC increased to 0.81 (95% CI 0.75-0.87) (black line).

## Supplementary Fig S8

Exploratory post-hoc analyses of patients with T3-4- (panel A and B) or T4-tumors (panel C and D) and outcome after intensified radiotherapy (either 1.1Gy+2.0 Gy per day, total dose 68.0 Gy or 2.15 Gy per day, total dose 73.1 Gy) compared with conventional fractionation (2.0 Gy per day, total dose 68.0 Gy).

For patients with T3-4 tumors, progression-free survival (panel A) with intensified RT (dotted line) was median 5.0 years (95% CI 4.6-NA) compared with 4.1 years (95% CI 2.3-NA) for standard RT (solid line). Corresponding numbers for overall survival (B) were 8.3 years (95% CI 4.9-NA) and 6.3 years (4.2-NA), respectively.

For patients with T4 tumors, progression-free survival (panel C) with intensified RT (dotted line) was median 4.9 years (95% CI 2.7-NA) compared with 2.2 years (95% CI 1.6-NA) for standard RT (solid line). Corresponding numbers for overall survival (D) were 7.6 years (95% CI 4.9-NA) and 3.6 years (2.8-6.6), respectively.

A)

B)

C)

D)
